# Vitamin-D deficiency predicts infections in young north Indian children: A secondary data analysis

**DOI:** 10.1371/journal.pone.0170509

**Published:** 2017-03-08

**Authors:** Ranadip Chowdhury, Sunita Taneja, Nita Bhandari, Bireshwar Sinha, Ravi Prakash Upadhyay, Maharaj Kishan Bhan, Tor A. Strand

**Affiliations:** 1 Centre for Health Research and Development, Society for Applied Studies, New Delhi, India; 2 Indian Institute of Technology - Delhi, New Delhi, India; 3 Knowledge Integration and Translational Platform (KnIT), Biotechnology Industry Research Assistance Council (BIRAC), New Delhi, India; 4 Centre for Intervention Science in Maternal and Child Health, Centre for International Health, University of Bergen, Bergen, Norway; 5 Department of Research, Innlandet Hospital Trust, Brumunddal, Norway; TNO, NETHERLANDS

## Abstract

**Background:**

Recent studies have demonstrated a relationship between poor vitamin D status and respiratory infections and diarrhea among young children. Acute lower respiratory infections (ALRI) and diarrhea are among the two most important causes of death in under-5 children. In this paper, we examined the extent to which vitamin-D deficiency (<10 ng/ml) predicts ALRI, clinical pneumonia and diarrhea among 6 to 30 months old children.

**Methods:**

We used data from a randomized controlled trial (RCT) of daily folic acid and/or vitamin B12 supplementation for six months in 6 to 30 months old children conducted in Delhi, India. Generalized estimating equations (GEE) were used to examine the associations between vitamin-D deficiency and episodes of ALRI, clinical pneumonia and diarrhea.

**Results:**

Of the 960 subjects who had vitamin-D concentrations measured, 331(34.5%) were vitamin-D deficient. We found, after controlling for relevant potential confounders (age, sex, breastfeeding status, wasting, stunting, underweight, anemia status and season), that the risk of ALRI was significantly higher among vitamin-D deficient (OR 1.26; 95% CI: 1.03 to 1.55) compared to vitamin-D-replete children in the six months follow-up period. Vitamin-D status was not associated with episodes of diarrhea or clinical pneumonia.

**Conclusion:**

Vitamin-D deficiency is common in young children in New Delhi and is associated with a higher risk of ALRI. The role of vitamin D in Indian children needs to be elucidated in further studies.

## Introduction

Vitamin D deficiency is considered to be the most common nutritional deficiency and often one of the most commonly undiagnosed medical conditions in the world [1]. The prevalence of vitamin D deficiency in young children is around 50–90% in the Indian subcontinent [2]. Vitamin D is primarily produced in the skin after exposure to ultraviolet radiation and less than 10% is derived from dietary sources [3].

Vitamin D is a potent immune-modulator of adaptive and innate immune responses [4]. In vitro studies have shown that 1,25-dihydroxyvitaminD3, the active metabolite of vitamin D, is important for promoting and regulating immune responses [5,6]. Observational studies suggest a link between low vitamin D concentrations and an increased risk of lower and upper respiratory tract infections in infants and young children [7]. A recent prospective cohort study found that vitamin-D deficiency was associated with increased rates of diarrheal illnesses among school-aged children [8]. However, the extent to which vitamin D deficiency predicts these infections in young children is less clear.

The estimated incidence of pneumonia in children under 5 years is 0.29 episodes per child-year in developing countries, resulting in 151 million new episodes each year, of which 7–13% of cases are severe enough to be life-threatening and necessitate hospital admission [9]. In 2013, 25.3% of deaths in children aged 1–59 months in India were due to pneumonia, totaling 150,169 deaths [10]. Globally, diarrhea causes 9% of all under-5 deaths, most of these in developing countries [10]. Although a reduction has been observed in the incidence of diarrhea in resource-limited settings, the disease burden associated with recurrent enteric illnesses still remains a public health problem [11–12] that results in excess childhood mortality [13–15].

Several micronutrients are important for innate and adaptive immunity in young children [16]. Recent meta-analyses have demonstrated a 15% reduction in childhood diarrheal incidence following vitamin-A supplementation [17] and 13% reduction in diarrheal and pneumonia incidence following zinc supplementation [18, 19]. The role of Vitamin-D as an immune-modulator has led to an increased interest in investigating its function in infectious diseases [20].

We conducted a randomized controlled trial (RCT) where children aged 6 to 30 months were supplemented daily with folic acid and/or vitamin B12 for six months. The main outcomes were the incidence of respiratory infections (ALRI, clinical pneumonia) and diarrhea. Enrolled participants were followed biweekly for respiratory and diarrheal morbidity [21]. Using data from this study we examined the extent to which vitamin-D deficiency (<10 ng/ml) at baseline predicted these outcomes during the 6 months follow-up period.

## Materials and methods

### Subject

The study was conducted from January 2010 to February 2012 in the low-to-middle socioeconomic settings of Tigri and Dakshinpuri in New Delhi. The total population of this site was about 300,000. Details of the population have been described previously [21]. This randomized double-blind placebo-controlled trial (NCT00717730 at www.clinicaltrials.gov) with a factorial design enrolled 1000 children, and evaluated the impact of supplementation with folic acid, vitamin B12, or both on childhood infections [21]. The analyses in the current manuscript are restricted to the group of 960 children whose baseline vitamin-D levels were available.

### Definitions

Diarrhea was defined as the passage of 3 or more loose or watery stools in a 24-h period. Two episodes of diarrhea were separated by a 72-hour or more diarrhea free period. ALRI was defined as cough or difficult breathing with an elevated respiratory rate above the age-specific cutoff values (≥ 50 breaths/min in infants and ≥ 40 breaths/min in older children) according to WHO criteria, or cough or difficult breathing and lower chest in drawing [22]. Clinical pneumonia was defined either by a combination of cough with crepitations or bronchial breathing by auscultation or as an episode of ALRI associated with at least one of the following features: lower chest indrawing, convulsions, inability to drink or feed, extreme lethargy, restlessness or irritability, nasal flaring, or abnormal sleeping and difficulty in waking.

### Analytical procedures

Blood samples were obtained at baseline from all the children; 3 mL blood was collected in an evacuated tube containing EDTA (Becton Dickinson). The plasma was centrifuged at ~ 450 × g at room temperature for 10 min, separated, and transferred into storage vials and stored at– 20°C until analyzed. Plasma concentration of vitamin-D was measured by quantitative electro-chemiluminescence binding assay, with detection of 25(OH) D_2_, the hydroxylated forms of vitamin D2 (Roche Diagnostics, Mannheim, Germany) [23] at Christian Medical College, Vellore biochemistry laboratory.

### Ethics

This study was conducted according the guidelines laid down in the Declaration of Helsinki. All procedures were approved by the Ethics committees of the Society for Applied Studies, New Delhi, Christian Medical College Vellore and Norwegian Regional Committee for Medical and Health Research Ethics (REK VEST). The consent form for the main trial also sought permission from parents to store these children’s blood specimen for use in future research. All parents consented for the same.

### Statistical analysis

Proportions and means (SD) or median (IQR) were calculated for categorical and continuous variables by Vitamin-D status at baseline. Vitamin D deficiency was defined at <10ng/mL (25 nmol/L) [24]. The 6 months’ follow-up period was divided into 26 periods of 7 d for every child. For a period to be included in the analyses, we required information on 4 d or more days of the given 7 d period. To account for interdependence of multiple observation periods in the same child, we used generalized estimating equations (GEE) with an autoregressive covariance-variance matrix taking time into account. In these models, occurrence of a new episode of diarrhea, ALRI, or clinical pneumonia in a child period was modeled as dependent variables and baseline vitamin D status as an independent variable. We included types of intervention received and other baseline variables as independent variables (age, sex, breastfeeding status, wasting, stunting, underweight, anemia status and season) in the model to adjust for potential confounding. The model used a logit link, binomial variance, autoregressive correlation and robust standard error to yield odds ratio (OR). We used STATA version 14 (Stata Corporation, College Station, TX) for most statistical analyses. We used generalized additive models in the statistical software R version 3.1.2 (The R Foundation for Statistical Computing, Vienna, Austria) to explore nonlinear associations between the vitamin D level at baseline and ALRI incidence after adjustment for potential confounders [25]. We also used generalized additive models to explore nonlinear associations between vitamin D level at baseline and season defined by period of enrollment in weeks. We considered an association to be statistically significant when the P value was <0.05. Post-hoc calculations of statistical power showed that we had more than 90% power to detect at least 25% more episodes of ALRI during 6 months follow-up in the vitamin- D deficient group compared to vitamin-D non deficient group with the available sample, at 5% significance level.

## Results

A total of 1000 children were included in the main trial. Blood samples for vitamin D were collected at baseline for 960 (96%) children. Of these, 331 (34.5%) children were Vitamin D deficient (<10 ng/ml). The baseline characteristics of the population by deficiency status are presented in Table 1. Approximately half of the enrolled children were boys and almost all (98%) were ever breast fed. Over 36.4% of the children were stunted, 31% underweight, and 10.7% wasted. Approximately 70% of the children were anemic.

**Table 1:** Baseline characteristics of Vitamin D deficient and non deficient children aged 6–30 months included in the analysis.

Characteristics	n = 960	
**Proportion of children**		
**Deficient (< 10 ng/ml)**	331 (34.5)	
**Non deficient (≥ 10 ng/ml)**	629 (65.5)	
	**Deficient**	**Non deficient**
	**n = 331**	**n = 629**
**Infant characteristics**		
Age at enrollment in months, mean (SD)	16.9 (7.1)	15.8 (7.0)
Proportion of children		
<12 months	91 (27.5)	210 (33.4)
12 to 23 months	166 (50.1)	306 (48.7)
24 to 30 months	74 (22.4)	113 (18.0)
Boys	162 (48.9)	328 (52.2)
Ever breastfed	325 (98.2)	622 (98.9)
Prevalence of illness in previous 24 hours		
Diarrhea	17 (5.1)	32 (5.1)
Cough or difficult breathing or fast breathing	114 (34.4)	192 (30.5)
**Anthropometric status**		
Mean(SD): Z score		
Weight for Height Z score(WHZ)	-0.86 (0.92)	-0.89 (0.94)
Height for Age Z score (HAZ)	-1.56 (1.24)	-1.63 (1.16)
Weight for Age Z score (WAZ)	-1.46 (1.06)	-1.52 (1.05)
Wasted (<-2 WHZ)	35 (10.6)	68 (10.8)
Stunted (<-2 HAZ)	117 (35.3)	233 (37.0)
Underweight (<-2 WAZ)	101 (30.5)	197 (31.3)
Anemia (Hb<11g/dl)	244 (73.7)	424 (67.4)
**Socio-demographic characteristics**		
Mother’s age in years, mean (SD)	26.3(5.8)	25.6(4.1)
Mother’s schooling in years, median (IQR)	8(5,10)	7(0,10)
Father’s schooling in years, median (IQR)	10 (7,12)	9 (6,12)
Annual family income in rupees, median (IQR)	72000 (60000–144000)	84000 (60000–138000)

Figures are number (percentage) unless stated otherwise

**Fig 1:**
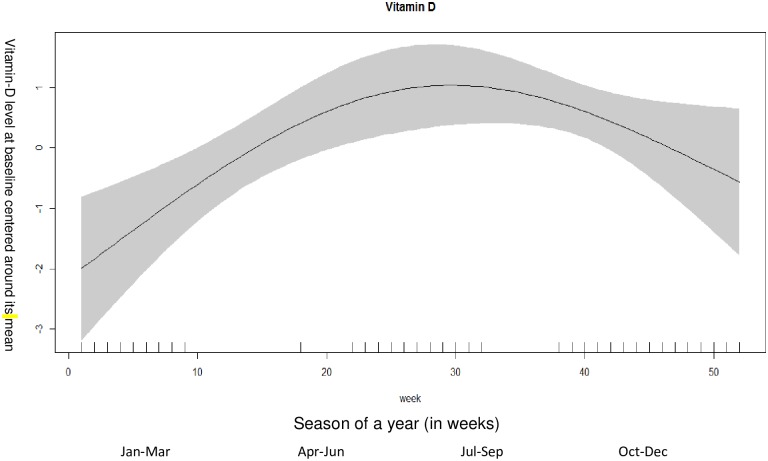
Associations between vitamin-D level at baseline and weeks of a year (among 960 children). The graph was constructed using generalized additive models in R, the solid line depicts the association of vitamin-D level at baseline and season. The shaded area spans the 95% confidence interval of this association.

Fig 1 shows the relationship between vitamin-D according to weeks of enrolment. As distinct seasons are difficult to define in India, we divided the period of enrollments into weeks. The vitamin D concentrations were higher in the 24^th^ to 32^nd^ weeks which correspond to months (May- July) with more daylight. The baseline vitamin D levels were lower for children who were enrolled in the initial weeks of the year which correspond to months (January-February) and have less daylight.

The diarrheal episodes in vitamin-D deficient and non-deficient children are shown in Table 2. There was no association between vitamin-D status and episodes of diarrhea overall and according to episodes of diarrhea lasting 6 days or more. The association between vitamin-D status and ALRI and clinical pneumonia are shown in Table 3. The incidence of ALRI was significantly higher among vitamin-D deficient children than in vitamin-D replete children (OR: 1.26; 95% CI: 1.03–1.55). However, the incidence of clinical pneumonia was not significantly associated with vitamin D status (OR: 1.05; 95% CI: 0.79–1.38).

**Table 2:** Incidence of diarrheal episodes in vitamin-D deficient and non deficient children.

	Deficient	Non Deficient	OR (95% CI)^b^
	(Vitamin D level < 10 ng/ml)	(Vitamin D level ≥ 10 ng/ml)^a^	
Total child-years of follow-up	162.3	308.5	
Episodes of diarrhea	775	1385	
Incidence density of diarrhea per child year (95% CI)	4.78 (4.44 to 5.12)	4.49 (4.26 to 4.73)	1.07 (0.95 to 1.20)
Episodes of diarrhea lasting			
> = 3 d	328	568	1.07 (0.96 to 1.19)
> = 5 d	182	339	0.98 (0.83 to 1.15)
> = 7 d	114	218	0.95 (0.76 to 1.20)
> = 14 d	34	63	1.01 (0.63 to 1.61)
Episodes of diarrhea with > = 6 stools/on any day	200	409	1.01 (0.83 to 1.22)

^a^ Reference category: Non Deficient (Vitamin D level ≥ 10 ng/ml)

^b^ ORs were calculated by using generalized estimating equations with a logit link, binomial variance, autoregressive correlation and robust standard error and adjusted for age, sex, breastfeeding status, wasted, stunted, underweight, anemia status, season and type of interventions

**Table 3:** Incidence of ALRI and clinical pneumonia in vitamin-D deficient and non deficient children.

	Deficient	Non Deficient	OR (95% CI)^b^
	(Vitamin D level < 10 ng/ml)	(Vitamin D level ≥ 10 ng/ml)^a^	
Total child-years of follow-up	162.3	308.5	
Episodes of ALRI	244	418	
Incidence density of ALRI per child year (95% CI)	1.50 (1.32 to 1.70)	1.35 (1.23 to 1.49)	1.26 (1.03 to 1.55)
Episodes of clinical pneumonia	144	294	
Incidence density of clinical pneumonia per child year (95% CI)	0.89 (0.75 to 1.04)	0.95 (0.85 to 1.07)	1.05 (0.79 to 1.38)

^a^ Reference category: Non Deficient (Vitamin D level ≥ 10 ng/ml)

^b^ ORs were calculated by using generalized estimating equations with a logit link, binomial variance, autoregressive correlation and robust standard error and adjusted for age, sex, breastfeeding status, wasted, stunted, underweight, anemia status, season and type of interventions

ALRI, acute lower respiratory infection

**Fig 2:**
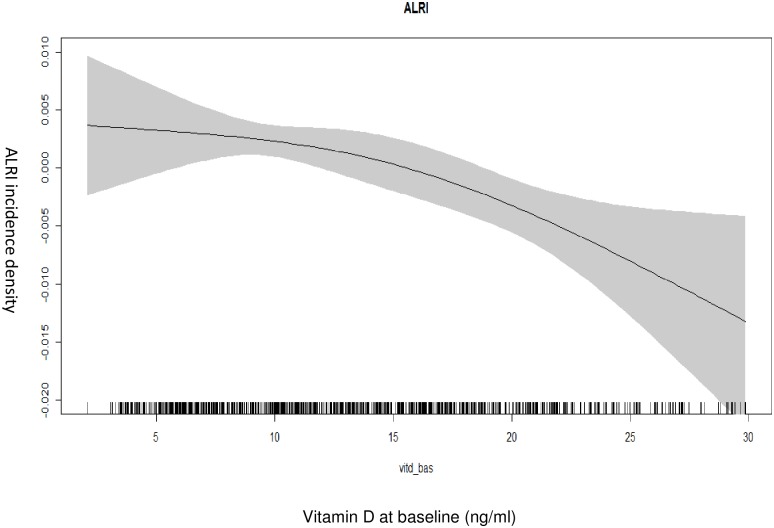
Associations between vitamin-D level at baseline and ALRI incidence density (among 960 children). The graph was constructed using generalized additive models in R, the solid line depicts the association of vitamin-D level at baseline and ALRI incidence density. The shaded area spans the 95% confidence interval of this association.

The association between baseline vitamin-D levels and incidence density of ALRI is depicted in Fig 2. The ALRI incidence density increases with decreasing baseline vitamin-D concentrations.

## Discussion

We report the prevalence of vitamin D deficiency and its association with common infections in young children. We found a high prevalence of vitamin D deficiency which is consistent with other studies in India [2]. However, a recent study from Nepal found that only <5% of breastfed infants were vitamin-D deficient, even when a higher cut off (<20 ng/l) was used [26]. High prevalence of vitamin-D deficiency observed in our study setting, in spite of abundant sunlight may be because of relatively high solar zenith angle, in combination with atmospheric pollution, type- V skin types of the population and restricted outside activities [27, 28]. More Ultraviolet B (UVB) photons are absorbed by the stratospheric zone, and therefore fewer UVB photons penetrate to earth’s surface to produce cutaneous pre-vitamin D3 with a relatively high solar zenith angle. [29]. A recent study indicated that infants may get enough vitamin D from breast milk if their mothers take high-dose vitamin D supplements [30]. The complementary foods in the diets of Indian infants and children are primarily cereal based and low in vitamin D [31]. This is probably another contributing factor of the widespread vitamin-D deficiency among children in this setting.

We found a significantly higher incidence of ALRI among vitamin-D deficient children when compared to vitamin-D replete children. Similar findings have been shown in previous observational studies [32–34]. Vitamin-D induces TLR activation and antibacterial responses which in turns enhances production of cathelicidin (LL-37), an endogenous antimicrobial peptide which is highly expressed at natural barrier sites e.g. lungs [35]. The protective role of vitamin-D against ALRI can be explained through its modulatory effect of both innate and adaptive immunity and regulatory function of inflammatory cascade [36–39].

We did not find any association with clinical pneumonia, a severe form of ALRI, and vitamin D status. Other observational studies have shown mixed results; while some studies found associations between vitamin-D status and clinical pneumonia, others did not [40–43]. Vitamin D has distinct effects on the innate and adaptive immune responses that may explain different roles in pathogen-specific infection severity [44]. Furthermore, a recent systematic review concluded that there was no evidence of vitamin D supplementation among under-5 children in the management of clinical pneumonia [45]. Even if vitamin D has a role in the defense against infections it might not have a therapeutic role as once an infection has taken place, other factors determine its course and how quickly it will resolve. Given the complexity of interaction of vitamin-D with the immune system and inflammatory cascade, more research is needed to further define the specific role of vitamin D in enhancing immune function and reducing the severity of infections.

Poor vitamin D status was not associated with an increased incidence and severity of diarrhea in our study, which is in line with findings from another observational study [46]. A randomized controlled trial with 3-monthly bolus supplementation with 100,000 IU of vitamin D3 among children aged 1 to 29 months, showed no effect on diarrheal illnesses [47]. Our study was done in urban slum where constant exposure to pathogenic organisms and subsequent enteric infection is common in children [48]. This could masquerade the potential beneficial role of vitamin-D in this population.

There may be deficiencies of other limiting micronutrients. Zinc deficiency increases the risk of diarrhea and pneumonia. A previous study in this population showed that zinc deficiency is common and zinc supplementation reduces the burden of diarrhea and lower respiratory tract infections [49,50]. It has been shown that vitamin-D-depenent genes in the cell are influenced by the intracellular zinc concentration [51]. Because the sources of vitamin D and zinc are different, we do not believe that vitamin D status is confounded by zinc status. However, there is a possibility that these nutrients may interact with each other.

The strengths of our study are that the data are from a well conducted study with very low attrition rates. We undertook multiple follow up visits for assessing ALRI, clinical pneumonia and diarrhea to ensure that virtually all episodes were documented. Outcomes were clearly defined and assessed by highly trained field staff. Results were adjusted for several relevant confounders including nutritional status of children and the season of enrollment.

We used an immunological method to measure vitamin-D concentration. It should be noted that immunoassays can overestimate 25OHD [52] because it is lipophilic which makes it vulnerable to matrix effects in the protein binding assays [53].

The results of this study could have important public health implications. As fortified foods have been recognized as an important source of vitamin D [54] such as oils, cereal powders and even salt supplementation and fortification may help in preventing vitamin D deficiency. Vitamin D supplementation is recommended in many countries, such public health interventions need serious consideration in the Indian context.

## Conclusion

The present study demonstrates that vitamin D deficiency is common in New Delhi children aged 6–30 months and that it is associated with increased risk of ALRI. Randomized controlled trials measuring the effect of vitamin D supplementation in these setting should be prioritized.
